# Supplementary material for: Potential Dietary and Therapeutic Strategies Involving Indole-3-Carbinole in Preclinical Models of Intestinal Inflammation
Source: Nutrients. 2023 Nov 30;15(23):4980. doi: 10.3390/nu15234980 (PMC10708520; doi:10.3390/nu15234980)
Supplement: Supplementary file 1 [file nutrients-15-04980-s001.zip › supplementary figures.pdf]

SUPPLEMENTRY FIGURE S1.

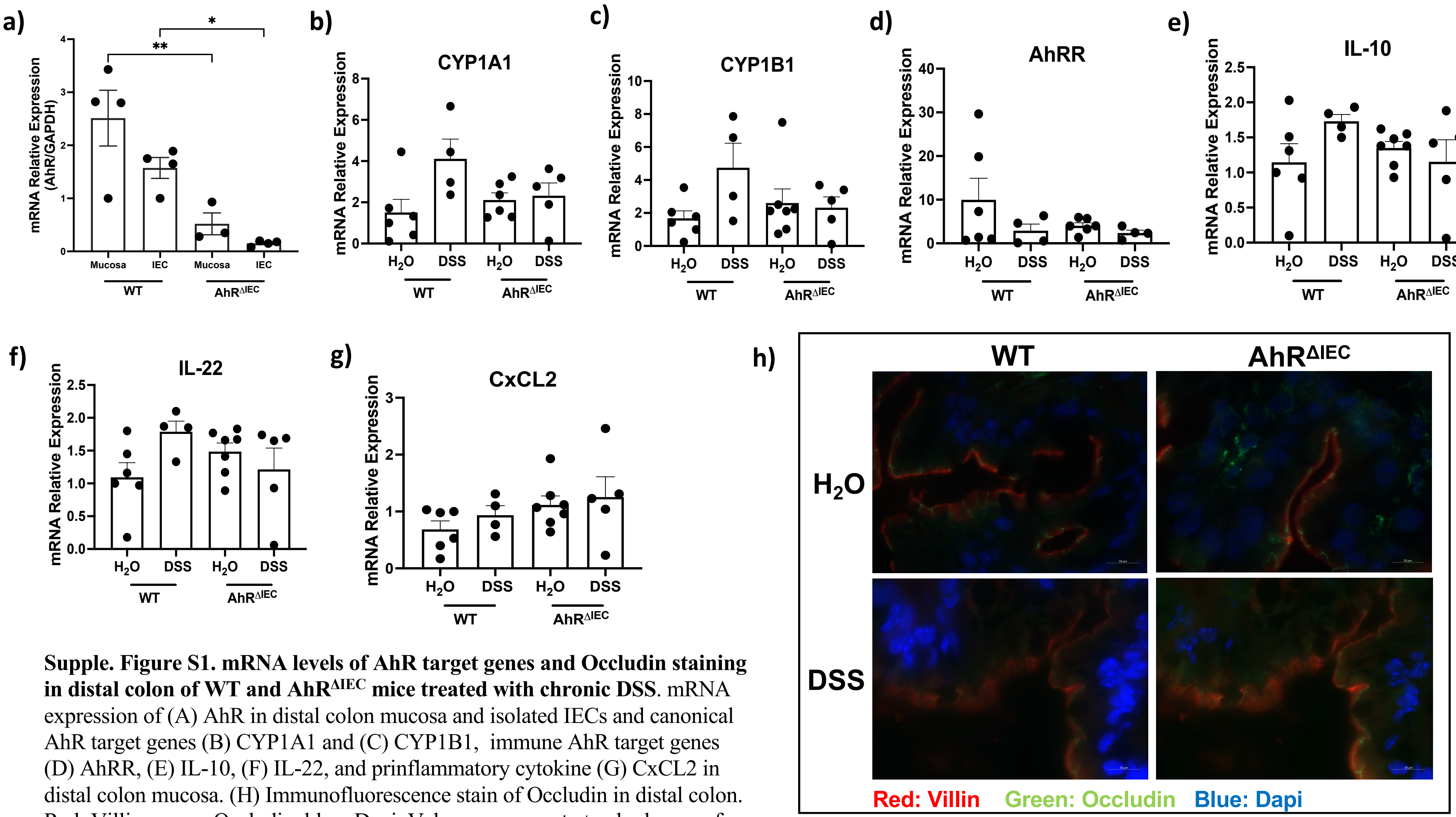

Supple. Figure S1. mRNA levels of AhR target genes and Occludin staining in distal colon of WT and AhR$^{\Delta IEC}$ mice treated with chronic DSS. mRNA expression of (A) AhR in distal colon mucosa and isolated IECs and canonical AhR target genes (B) CYP1A1 and (C) CYP1B1, immune AhR target genes (D) AhRR, (E) IL-10, (F) IL-22, and prinflammatory cytokine (G) CxCL2 in distal colon mucosa. (H) Immunofluorescence stain of Occludin in distal colon. Red: Villin, green: Occludin, blue: Dapi. Values are mean ± standard error of mean. Data. Values are mean ± standard error of mean. Data were analyzed by One-Way ANOVA. ** $P < 0.05$, ** $P < 0.01$,

SUPPLEMENTRY FIGURE S2.

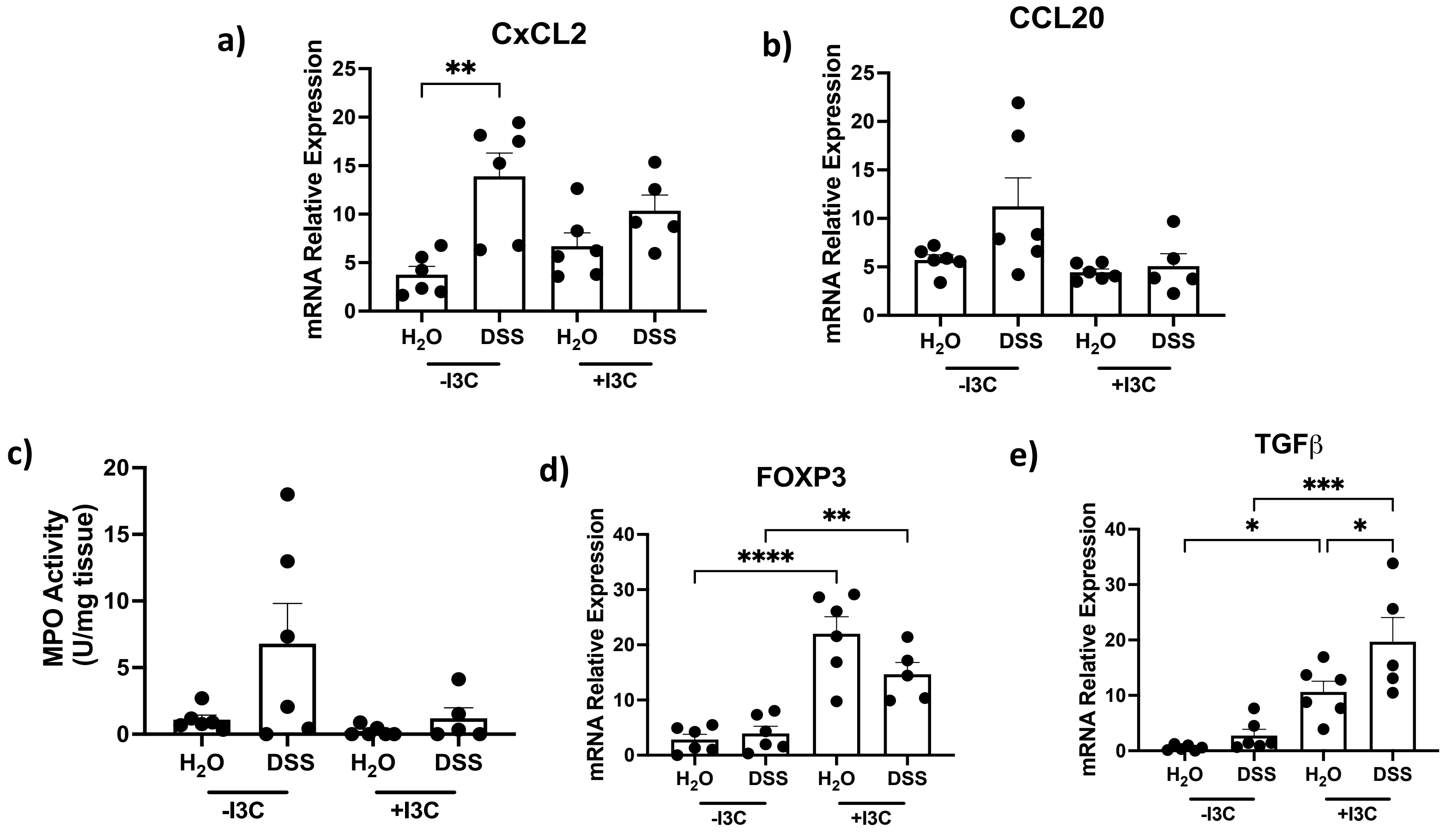

Supple. Figure S2. Inflammation in WT mice treated with chronic DSS and fed -I3C or +I3C diets. mRNA levels of (A) CXCL2 and (B) CCL20 in distal colon. Levels of reactive oxygen species (ROS) was measured by (C) MPO activity. mRNA expression of (D) FOXP3 and (E) TGFβ in the spleen of WT and $AhR^{\Delta IEC}$ mice treated with DSS. Data were analyzed by One-Way ANOVA. ** $P < 0.01$, **** $P < 0.0001$.

SUPPLEMENTRY FIGURE S3.

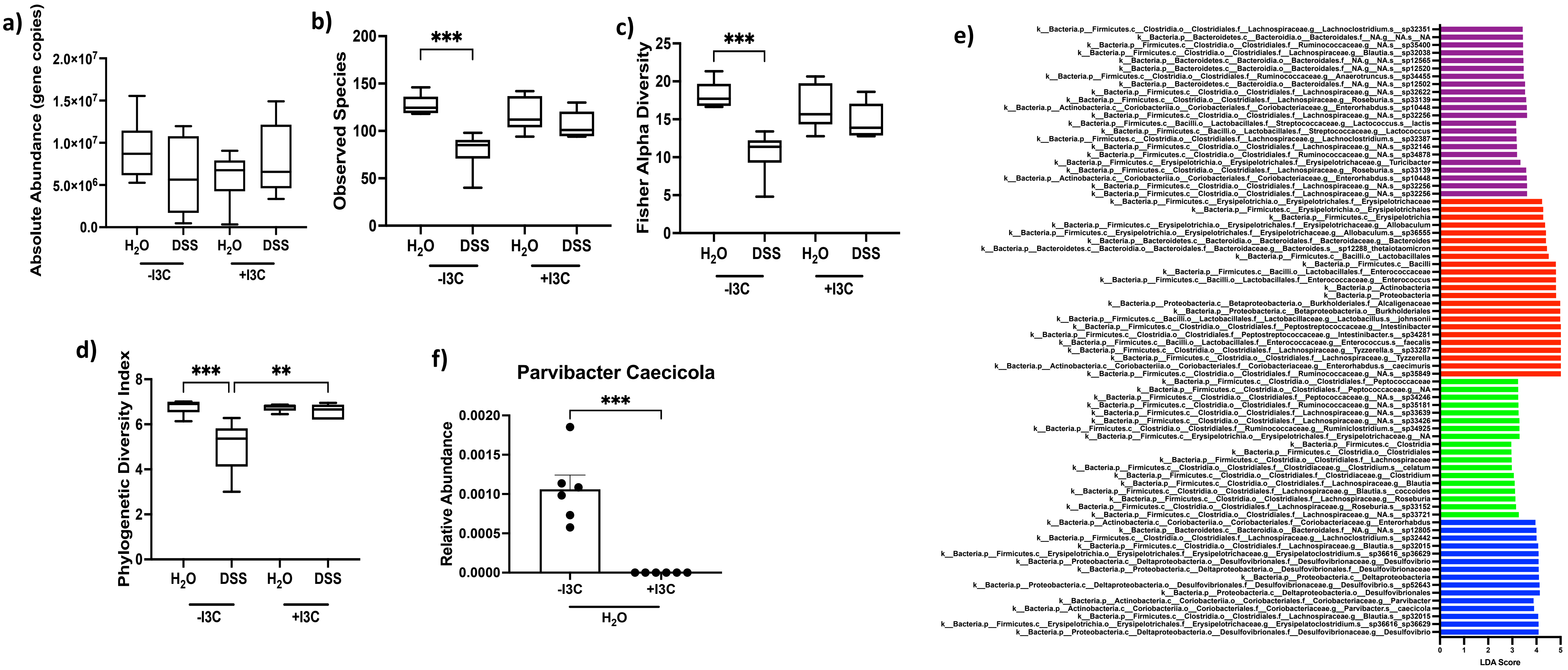

Supple. Figure S3. Microbiota analysis of WT mice fed -I3C or +I3C and induced with chronic colitis. (A) Absolute abundance of microbial species in WT mice induced with chronic colitis and fed -I3C or +I3C diets. Alpha diversity assessment by (B) observed species, (C) fisher alpha diversity and (D) phylogenetic diversity index. (E) LefSe analysis of top abundantly different species. (F) Relative abundance of *Parvibacter caecicola* in WT mice fed -I3C or +I3C diets. Values are mean ± standard error of mean. Data were analyzed by Zymo MicroBiomics and One-Way ANOVA. * $P < 0.05$, ** $P < 0.01$, *** $P < 0.001$, **** $P < 0.0001$.

SUPPLEMENTRY FIGURE S4.

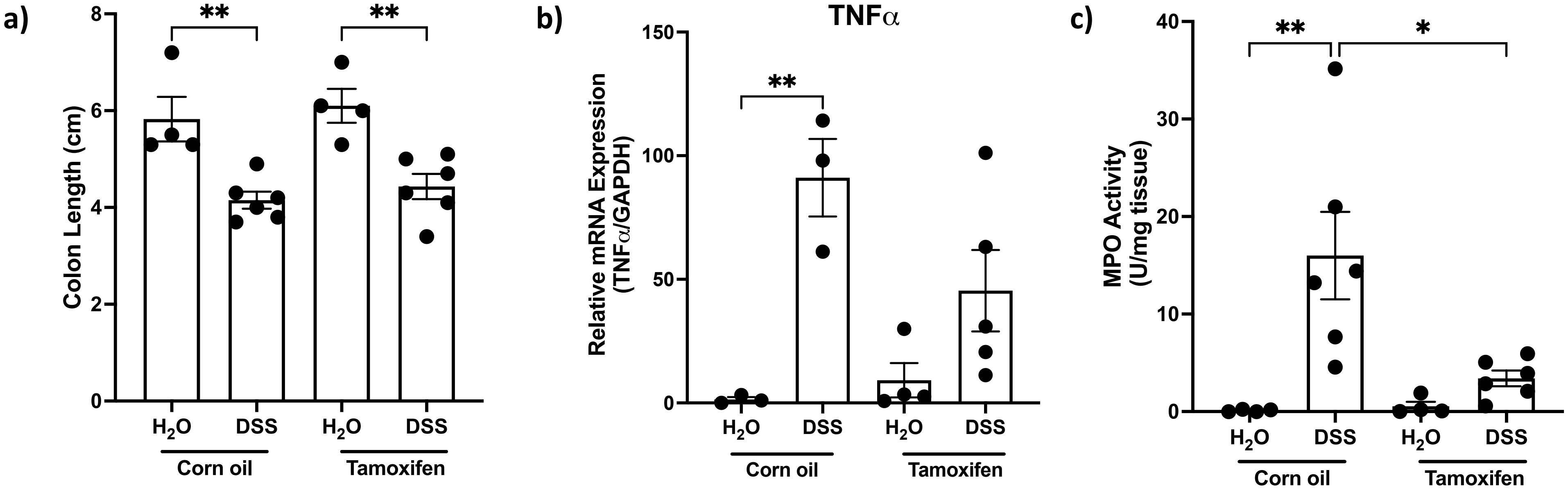

Supple. Figure S4. C57BL6/J mice treated with tamoxifen or corn oil and induced with DSS-colitis. C57BL6/J were purchased from Jackson Laboratory at 8 weeks of age. These mice received IP tamoxifen or the vehicle control injections for five consecutive days and were subsequently induced with DSS-colitis. (A) Quantification of colon lengths, (B) mRNA levels of TNFα in the distal colon mucosa, and (C) MPO activity in the distal colon. Values are mean ± standard error of mean. Data were analyzed by One-Way ANOVA. * $P < 0.05$, ** $P < 0.01$.

SUPPLEMENTRY FIGURE S5.

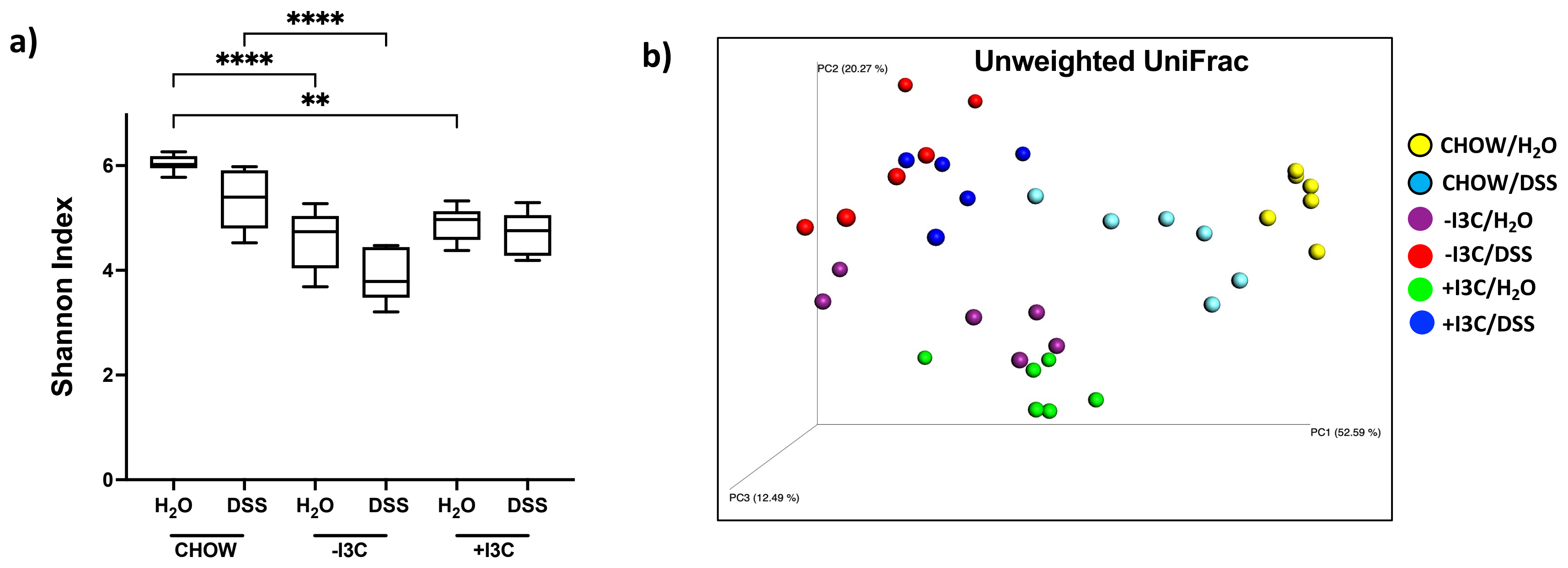

Supple. Figure S5. Microbiota analysis of WT mice fed Chow, -I3C, or +I3C and induced with chronic colitis. Alpha diversity was assessed by (A) Shannon index and beta diversity was assessed by (B) Unweighted UniFrac. Values are mean ± standard error of mean. Data were analyzed by Zymo MicroBiomics and One-Way ANOVA. ** $P < 0.01$, **** $P < 0.0001$.
